# Cancer cachexia as a multiorgan failure: Reconstruction of the crime scene

**DOI:** 10.3389/fcell.2022.960341

**Published:** 2022-09-08

**Authors:** Michele Ferrara, Maria Samaden, Elena Ruggieri, Emilie Vénéreau

**Affiliations:** ^1^ Tissue Regeneration and Homeostasis Unit, Division of Genetics and Cell Biology, IRCCS San Raffaele Scientific Institute, Milan, Italy; ^2^ Vita-Salute San Raffaele University, Milan, Italy

**Keywords:** cancer cachexia, immune system, skeletal muscle, adipose tissue, liver, pancreas, gut, brain

## Abstract

Cachexia is a devastating syndrome associated with the end-stage of several diseases, including cancer, and characterized by body weight loss and severe muscle and adipose tissue wasting. Although different cancer types are affected to diverse extents by cachexia, about 80% of all cancer patients experience this comorbidity, which highly reduces quality of life and response to therapy, and worsens prognosis, accounting for more than 25% of all cancer deaths. Cachexia represents an urgent medical need because, despite several molecular mechanisms have been identified, no effective therapy is currently available for this devastating syndrome. Most studies focus on skeletal muscle, which is indeed the main affected and clinically relevant organ, but cancer cachexia is characterized by a multiorgan failure. In this review, we focus on the current knowledge on the multiple tissues affected by cachexia and on the biomarkers with the attempt to define a chronological pathway, which might be useful for the early identification of patients who will undergo cachexia. Indeed, it is likely that the inefficiency of current therapies might be attributed, at least in part, to their administration in patients at the late stages of cachexia.

## Introduction

Cancer cachexia is a severe co-morbidity affecting about 50–85% of cancer patients, according to cancer type and stage.(Argilés et al., 2014; Argilés et al., 2019a; Biswas and Acharyya, 2020). It is characterized by unintentional weight loss due to skeletal muscle wasting, and frequently loss of fat mass, leading to death in 20–30% of all cancer patients (Argilés et al., 2019a). In contrast to malnutrition or starvation, the simply nutritional supports are largely inefficacious, highlighting the multifactorial aetiology of the syndrome. Despite the high incidence of cachexia among cancer patients, drugs directly targeting this syndrome are still lacking, making it an urgent medical need, and nowadays it is largely accepted that only a multimodal approach will be successful in the management of cachexia. Over the years, a large knowledge has been obtained but cancer cachexia remains elusive in many aspects. Although the widespread manifestation of this syndrome and its severe impact on quality of life of both patients and their family, therapeutic opportunities are not available yet, and clear diagnostic criteria and biomarkers are still away from clinical practice. Indeed, the main focus frequently remains to fight only tumor progression, with the consequence of ruling out sometimes other critical symptoms by passively accepting them as “epiphenomena,” as cachexia was considered in the past. However, the clinical scenario is far more complex, and to distinguish cachexia from age-related muscle wasting (sarcopenia) and malnutrition is really challenging in the majority of multi-affected elderly patients. A global effort will be crucial in the long pathway toward an efficacious management of cancer cachexia. The goal of this review is to summarize the key findings in tissues affected by cancer cachexia and to discuss the temporal evolution of multiple organ dysfunction observed in this syndrome, with final remarks on critical issues that should be addressed to counteract cachexia in patients.

## Definition of cachexia and similarities/differences with other muscle wasting disorders

A clinical consensus (Fearon et al., 2011) describes cachexia as an unintentional body weight loss of more than 5% over the past 6 months. Although cachexia proceeds continuously, a chronological path identifying three different subgroups have been defined: pre-cachexia, cachexia, and refractory cachexia. The latter stage is associated with no response to anticancer therapies and a low performance status, with a life-expectancy of less than 3 months (Fearon et al., 2011). Despite the majority of studies focus on cachexia and refractory cachexia, the most intriguing phase is pre-cachexia, in which the advent of screening of reliable biomarkers, together with an efficacious therapeutic management, could really make the difference for patient. In this context, it is evident that a huge effort should be made to address the diagnosis of pre-cachexia in clinical context, in particular a general protocol should be established to screen cancer patients for the risk of developing cachexia.

The global consensus established in 2011 define pre-cachexia as an early phase in which metabolic derangements, such as impaired glucose tolerance and anorexia, are already present, but the weight loss is substantially absent (≤5%) (Fearon et al., 2011). Beside this clinical consensus, other classifications have been proposed (Argilés et al., 2017; Vigano et al., 2017; Argilés et al., 2019a). All these classifications discriminate between cachexia severity, but Argilés and collaborators proposed a Cachexia Score (CASCO) as a numerical score obtained from 5 different components (body weight loss and composition, inflammation/metabolic disturbances/immunosuppression, physical performance, anorexia, and quality of life), rendering the patient assignment to each class more feasible (Argilés et al., 2011; Argilés et al., 2017; Argilés et al., 2019b). Pre-cachexia, as defined before, is characterized by early cachexia-related derangement without weight loss. Therefore, pre-cachexia is diagnosed in CASCO approach by reaching a score >35 considering all the parameters but excluding the component of weight loss (Argilés et al., 2017). In a more recent paper, cancer patients without cachexia were stratified in different classes according to different risk to undergo cachexia (Vagnildhaug et al., 2019). The authors divided patients affected from different tumor types in five classes of increasing risk to undergo cachexia, according to clinical parameters, and were able to distinguish risk-level 1 patients, who did not reach the median time for cachexia onset, from risk-level 5 patients, who had 51 days as median time for cachexia manifestation. The latter class represents patients with 3–5% weight loss in the last 6 months, therefore very closed to the diagnosis of cachexia, consistent with short time measured for syndrome development (Vagnildhaug et al., 2019). Hence, this study uncovers interesting characteristics of the pre-cachexia phase, highlighting important predictors of cachexia development, such as cancer type, appetite, and initial weight loss.

In addition to the precise diagnosis of pre-cachexia phase, another critical issue is to distinguish between cachexia and other muscle wasting syndromes, such as sarcopenia and malnutrition, which have different molecular mechanisms and therefore therapeutic managements. The European Working Group on Sarcopenia in Older People 2 (EWGSOP2) defines sarcopenia as a muscle disease rooted in adverse muscle changes accruing across a lifetime, while malnutrition is the involuntary weight loss resulting from lack of nutrient intake or uptake (Meza-Valderrama et al., 2021). These three muscle wasting conditions share characteristics and unfortunately can also be present simultaneously. Sarcopenia, typically associated with aging, is a global and progressive muscle wasting disorder, which can be manifested alone (primary sarcopenia) or in association or not with inflammation and/or other diseases (secondary sarcopenia). In contrast, the underlying mechanism of malnutrition relies on a negative energy balance due to an inappropriate nutrient uptake and it strongly predicts severe sarcopenia. Cachexia is characterized by muscle wasting and negative energy balance and it is always associated with an inflammatory disorder but, in contrast to malnutrition and sarcopenia, cachexia cannot be reversed by simply nutritional or lifestyle supports (Meza-Valderrama et al., 2021).

It is crucial to note that, despite all tumors need substrates to grow and induce a systemic inflammation, not all the tumor types provoke muscle wasting, ranging from the most associated with cachexia, such as pancreatic cancer, to the only marginally affected, such as prostate and breast cancer (Baracos et al., 2018). Therefore, cachexia must be the result of a plethora of stimuli, produced by tumor cells and/or multiple tissues/organs, which act simultaneously and are not common to all cancer types. Finally, cancer refers to hundreds of different diseases and in the same way, cachexia has common characteristics but display specific manifestations according to patient background and tumor type. Due to the multifactoriality of cancer cachexia syndrome, *in vitro* studies are not sufficient to understand the global puzzle, although useful in certain conditions such as for the identification of catabolic pathways acting on skeletal muscle. Therefore, the majority of our knowledge on cancer cachexia rely on mouse pre-clinical models, which we discuss in the next section (for a complete review on the topic please refer to (Ballarò, Costelli and Penna, 2016; Penna, Busquets and Argilés, 2016).

## Cancer cachexia preclinical models

The widely employed subcutaneous transplantation of cancer cells, such as colon cancer C26 and Lewis lung carcinoma (LLC) cells, is a fast and practical model of cancer cachexia that generated most of our knowledge in the field.

In the C26 model, Balb/c or CD2F1 mice undergo body weight loss and muscle wasting, mainly through increased levels of circulating Interleukin-6 (IL-6), with premature death by 14–30 days from cell inoculation, according to the individual cell clones (Aulino et al., 2010; Talbert et al., 2014; Bonetto et al., 2016; Petruzzelli et al., 2022). Due to the high velocity and penetrance of cachexia induction in C26-bearing mice, this model is suitable for studies on cachexia *per se*. However, it is less suitable for the study of cachexia progression over time especially in case of very aggressive clones.

The other largely employed model is transplantation of Lewis Lung Carcinoma (LLC) cells in C57Bl/6 mice, the most suitable (almost unique) model for all the studies in genetically modified mice, such as knock-out and knock-in mice. LLC-derived tumors grow very fast and induce a systemic upregulation of Tumor Necrosis Factor-α (TNF-α). It takes more time to induce cachexia in LLC-bearing mice, and mice need to be euthanized by 35–40 days from tumor injection. There are other cancer cells inducing cachexia, such as melanoma B16 and adenocarcinoma MAC16, but they have been far less explored (Ballarò, Costelli and Penna, 2016). Finally, the use of human cells should be employed with care due to the necessity of transplantation in immunocompromised animals, thus lacking a complete immune response, one of the major components of cachexia. Beside models in mice, cancer cachexia has been also investigated in similar models in rat, such as Walker 256 carcinosarcoma and Yoshida ascites hepatoma 130 (AH130) (Ballarò, Costelli and Penna, 2016).

Compared to cancer cell inoculation, genetic models, such as APC^Min/+^ and KPP (K-Ras; p53; Cre pancreatic cancer) (Mehl et al., 2005; Talbert et al., 2019), are closer to human scenario, making them more suitable for translational research, but due to time, costs, and low penetrance, these models are far less employed. Apc^Min/+^ mice carry out a heterologous mutation in the Apc tumor suppressor gene, predisposing them to intestinal and colon tumor development (Rapaich Moser et al., 1990). So far, it is the most widely used genetic engineered mouse model to study cancer cachexia. These mice develop intestinal polyps by ∼4 weeks of age and loses body weight gradually between ∼14 and ∼20 weeks of age, reaching the peak of mortality at 20–24 weeks of age (Mehl et al., 2005).

The lack of valid therapeutic interventions after years of studies on cancer cachexia suggests that these pre-clinical models are essentially inadequate to recapitulate the human syndrome. Besides the kinetics of cachexia development, these models typically employ young mice, whereas in humans, cancer and cachexia are mainly associated with elderly patients. The age of mice seems to affect or not cachexia development according to the preclinical models, with no effect in C26-bearing mice (Talbert et al., 2014), while an influence of age has been reported in LLC model (Geppert et al., 2021), enforcing the concept that more attention should be paid to the age of the animals employed in preclinical models. However, the implementation of preclinical models is not obvious, due to the complexity of cachexia. Indeed, other comorbidities are often present simultaneously with cachexia in elderly patients affected by cancer, influencing its progression and generating confounding effects, as in the case of sarcopenia. Finally, cancer treatments (chemotherapy and radiotherapy) can lead to muscle wasting, worsening and confounding the scenario. For the latter reason, some investigators explored the molecular mechanisms underlying chemotherapy-induced cachexia (Le Bricon et al., 1995; Gilliam and Clair, 2011; Garcia et al., 2013; Chen et al., 2015; Damrauer et al., 2018; Breen et al., 2020; Conte et al., 2020), but these findings should be considered as a small piece of information in the complex puzzle of cancer cachexia.

In the next sections, we summarize some key findings on the deregulation of different tissues in cachexia. However, it is crucial to keep in mind that most of this knowledge is coming from studies performed in these preclinical models and in overt cachexia conditions. The lack of efficacious therapies against cachexia suggest that these approaches have been only partially helpful and that our comprehension of cachexia is still too fragmented (Roeland, 2021).

## Skeletal muscle: A main target but not the first affected tissue

Skeletal muscle is the biggest tissue in human body, accounting for about 40% of total body weight. Therefore, severe loss of muscle mass, as observed in cachexia, highly affects total body weight. In addition, muscle mass loss is coupled with reduced functionality, resulting in significant worsening of life quality and survival and, at the end-stage of disease, can lead to sudden death due to respiratory and/or cardiac failure (Argilés et al., 2019a). Most studies focus their attention on the molecular mechanisms leading to this unintentional muscle weight loss and numerous reviews have summarized the knowledge on this process (Fearon, Glass and Guttridge, 2012; Argilés et al., 2014; Cohen, Nathan and Goldberg, 2014; Baracos et al., 2018; Sartori, Romanello and Sandri, 2021). In brief, the loss of protein content in skeletal muscle is the result not only of the activation of several pro-catabolic stimuli (such as Interleukin-6, Interleukin-1, Tumor Necrosis Factor-α, Myostatin etc.) but also of the absence or reduction of pro-anabolic signals (such as Growth Hormone, Insulin, Insulin-like Growth Factor-1, sexual hormones etc.). Collectively, these stimuli lead to the activation of three main catabolic pathways: ubiquitin-proteasome system, autophagy and cathepsins (Porporato, 2016), finally resulting in an excess of protein degradation and thus loss of muscle mass. In this context, the availability of energetic substrates, such as aminoacids from muscles, fuels tumor growth both directly by acting on cancer cells and indirectly by sustaining gluconeogenesis in the liver (Porporato, 2016).

Among the common characteristics, muscle wasting is by definition the crucial issue of cachexia. In the clinical context, it is reasonable to hypothesize that pre-cachexia phase, which is the most associated with positive therapeutic options, display no muscle wasting. Indeed, patients with non-small cell lung cancer (NSCLC) at stages I-III display pre-cachexia as no changes in fat and lean body mass and any activation of ubiquitin-proteasome system are observed, despite the presence of systemic inflammation and a reduction in muscle performance (Op den Kamp et al., 2012). Lung cancer patients display elevated plasma levels of soluble TNF receptor 1 (sTNF-R1), fibrinogen, and C-reactive protein (CRP), as well as reduced albumin, highlighting sustained pro-inflammatory conditions. However, no inflammatory and catabolic pathways are activated in skeletal muscle at early stages, suggesting that only a prolonged exposure to a pro-inflammatory status and/or additional elements are required to induce muscle wasting (Op den Kamp et al., 2012). Accordingly, other studies reported similar results in lung and gastric cancer patients (Jagoe et al., 2002; Smith et al., 2011). Although pre-clinical models are distant from human scenario, the observation that inflammation and other derangements precede muscle wasting has been recapitulated in C26-bearing mice (Petruzzelli et al., 2022), in which spleen enlargement and adipose tissue loss occur before skeletal muscle atrophy. Similarly, splenomegaly precedes of at least 2 months muscle wasting in APC^Min/+^ mice (You et al., 2006).

Collectively, the studies on mouse models of cancer cachexia allow us to extract information and to understand when muscles start to break down. In murine models of tumor cells transplantation, it varies depending on cancer cells: at ∼15–18 days after LLC transplantion; at ∼10 or ∼21 days after C26 inoculation, according to different subclones. In the case of APCMin/+ mice, muscle wasting is evident starting from ∼16 to 18 weeks of age. In patients, the timeframe between tumor development and muscle wasting is obviously challenging to determine, due to multiple variabilities that cannot be taken into account.

In the context of muscle wasting, heart proteins can also be affected. Indeed, death in cachectic patients frequently occurs for respiratory failure or cardiac arrest, due to protein loss in diaphragm or heart, respectively (Nichols, Saunders and Knollmann, 2012). Symptoms frequently observed in patients with cachexia are fatigue, impaired exercise tolerance, short breath, all indicators of heart failure (Argilés et al., 2019a). It has been assumed that cardiac proteins were initially preserved and that their breakdown determines heart atrophy and failure only at late timepoints, worsening patients’ quality of life. However, heart weight has been correlated with Body Mass Index (BMI) in a retrospective analysis on patients affected by different cancer types (Barkhudaryan et al., 2017), suggesting that heart protein loss cannot be considered only an end-stage of cachexia development. Consistently, heart weight loss is frequently present when cachexia is fully established in pre-clinical models (Tian et al., 2011; Olivan et al., 2012). The underlying mechanisms seem to be related to the increase of both ubiquitin-proteasome and authophagy pathways in heart, resulting in cardiac derangements that finally lead to an increase in oxygen consumption and consequently energy expenditure, contributing to the negative energy balance, one of the hallmarks of cachexia syndrome (Argilés et al., 2019a).

In conclusion, skeletal muscle wasting, including heart weight loss, is not the first sign of cachexia development, although it is one of the main hallmarks. Indeed, cachexia represents a global tissue deregulation, beyond musle wasting (Wyart et al., 2020) and other tissues are affected before skeletal muscle, such as the immune system.

## Immune system: Friend and foe

The immune system is central in the cachectic process as it links tumor masses and all tissues and organs directly implicated in the progression of cachexia, such as adipose tissue, brain, liver, gut, or heart. It is now well established that systemic inflammation is a key driver of cancer cachexia, through circulating molecules released by immune cells that have direct effects on skeletal muscle (Baracos et al., 2018), and through the induction of other systemic disruptions that can in turn modulate skeletal muscle mass, such as the control of central nervous system, appetite, energy intake and expenditure, insulin resistance and hypogonadism (Laird and Fallon, 2017; Wu and Ballantyne, 2017; Baracos et al., 2018). Accordingly, preclinical and clinical studies have been conducted to evaluate whether physical exercise might improve muscle performance in patients with lung cancer, and they evidenced an interplay between physical exercise, the immune system and also the intestinal microbiota (Cortiula et al., 2022).

Tumor necrosis factor-α, also known as “cachectin”, has been the first cytokine identified to trigger cachexia mainly through its direct catabolic effect on skeletal muscle (Tracey, Lowry and Cerami, 1988). Interleukin-1β (IL-1β) and IL-6 have also been reported to be associated with the cachectic phenotype both in animal models and patients (Strassmann et al., 1993; Graziano et al., 2005; Zhang et al., 2007; Scheede-Bergdahl et al., 2012; Narsale and Carson, 2014; Pettersen et al., 2017). Neuroinflammation mediated by IL-1β results in increased muscle proteolysis and adipose lipolysis, which in turn leads to loss of appetite and increase of resting energy expenditure (Laird et al., 2021). In the skeletal muscle, IL-6 induces proteasome and autophagy protein degradation pathways that lead to wasting (Baltgalvis et al., 2008). In addition, IL-6 can also target adipose tissue, gut, and liver, evidencing its central role in cachexia (Narsale and Carson, 2014; Zimmers, Fishel and Bonetto, 2016). Beside their individual effects, these cytokines can cooperate to trigger several pathological mechanisms such as systolic heart failure (Lavine and Sierra, 2017), liver dysfunction (Seelaender et al., 1998; Jones et al., 2012; Gonçalves et al., 2019), bone loss (Mahon and Dunne, 2018) or mucosal damage and gut permeability (Costa et al., 2019). More recently, other cytokines have been identified as mediators of cancer-induced muscle wasting such as TNF-like inducer of apoptosis (TWEAK), TNF receptor (TNFR)-associated factor 6 (TRAF6), interferon gamma (IFN-γ), and leukemia inhibitory factor (LIF) (Smith et al., 2007; Kumar, Bhatnagar and Paul, 2012; Johnston et al., 2015; Kandarian et al., 2018). Interestingly, single nucleotide polymorphisms in the IL-1, IL-6 and IL-10 genes have been associated with cachexia in gastrointestinal cancers (Hishida et al., 2019). These findings suggest that genetic variation in immunity might be responsible for the predisposition of patients affected by the same cancer type to develop or not cachexia.

The identification of these cytokines as central players in cachexia led to the development of therapeutic strategies focusing on their targeting (Argilés, López-Soriano and Busquets, 2012). Clinical trials have been conducted to evaluate the therapeutic properties of thalidomide (a-N-phthalimidoglutaramide), etanercept and infliximab as TNF-α production suppressors (Gordon et al., 2005; Monk et al., 2006; Wiedenmann et al., 2008), and of monoclonal antibodies blocking IL-6 pathway (Rigas et al., 2010; Ando et al., 2014). Broad-spectrum peptide immunomodulator drugs have also been evaluated in clinical trials on cachectic patients, resulting in a good safety profile and improvement of body weight and physical performance (Argilés, et al., 2019b). Although these clinical trials have evidenced some therapeutic properties of these cytokine-directed strategies, the results were largely unsatisfactory for the management of cancer cachexia patients. Hence, a better understanding of the roles played by the different tissues and organs, including the immune system, and the inter-tissue crosstalk is essential for the development of effective therapeutic strategies. An example of this crosstalk in the context of cancer cachexia is the contribution of neutrophils infiltration and microglia activation to brain dysfunction (Burfeind et al., 2020; Kashihara et al., 2020).

Related to this, little is known on the role of the different leukocyte populations in the progression of cancer cachexia. A study reported a reduced number of macrophages and neutrophils in cachectic muscles of C26-bearing mice (Inaba et al., 2018). Conversely, a recent article reported that neutrophilia appears to be an early systemic event upon tumor growth and, notably, the number of neutrophils is also increased in lung and liver at early timepoint (Petruzzelli et al., 2022). The authors suggested that the systemic neutrophilia might originate from the spleen as increased spleen size and high number of splenic neutrophils progenitors were observed prior to the onset of the cachectic phenotype. Beside neutrophils number, their metabolism was affected in tumor-bearing mice with an overall increase in metabolism and dependence on glycolysis (Petruzzelli et al., 2022). Targeting of neutrophilia or aerobic glycolysis worsens the cachectic phenotype and the survival, indicating that neutrophilia might represent an adaptative response to preserve the systemic metabolic homeostasis during cancer progression (Petruzzelli et al., 2022). In addition, another study reported an early infiltration of neutrophils into regions of the brain that influence feeding behavior and/or energy metabolism, such as the hypothalamus, which contributes to anorexia and muscle atrophy in a mouse model of pancreatic ductal adenocarcinoma (Burfeind et al., 2020). Accordingly, it has been suggested that microglial cells in the brain might have a protective effect against severe cachexia by mediating depletion of neutrophils (Baazim, Antonio-Herrera and Bergthaler, 2022).

According to macrophages, it has been reported that in adipose tissue they contribute to the regulation of fat loss in a model of hepatocellular carcinoma (HCC)-associated cachexia (Erdem et al., 2019). Similarly, CD163^+^ macrophage muscle infiltration correlates with skeletal muscles atrophy in patients with pancreatic cancer and macrophage depletion leads to reduced systemic inflammation and muscle wasting in pancreatic tumor-bearing mice, indicating that both macrophage number and polarization might play a role in the progression of cancer cachexia (Shukla et al., 2020). A high number of Myeloid-derived suppressor cells (MDSCs), a heterogeneous population of myeloid cells with immunosuppressive functions, have been reported in tumours of patients affected by gastric and pancreatic cancers (Ohki et al., 2012; Khaled et al., 2014) and in several mouse models of cancer cachexia, such as C26 and LLC-bearing mice (Cuenca et al., 2014). MDSCs expansion in the tumour, bone marrow and spleen has been correlated with total body weight loss and modulation in energy metabolism, but the underlying mechanism remains to be defined (Cuenca et al., 2014).

The contribution of T-cells to the cachectic syndrome is also largely unknown. A study reported a positive relationship between the total number of T-cells, granulocyte/phagocytes, and CD3^−^CD4^+^ cells with muscle mass status in cancer patients, and gene correlation analyses indicate that the presence of CD8^+^ T-cells appears to be negatively correlated with the expression of key genes within muscle catabolism (Anoveros-Barrera et al., 2019). Similarly, significant correlations between frequencies of circulating T-cell populations and muscle strength, performance, and body mass have been reported in a small cohort of patients with gastrointestinal cancer (Narsale et al., 2019). In addition, the maintenance of body weight upon infection-associated cachexia in CD8^+^ T-cell null mice strongly suggest that CD8^+^ T-cells contribute to skeletal muscle wasting (Baazim et al., 2019). In a mouse model of cancer cachexia, the adoptive transfer of CD4^+^CD44Low naïve T-cells reduces muscle atrophy, muscle protein and DNA loss, even when inoculated after the onset of cachexia, associated with a protection from CD4^+^ T-cell lymphopenia (Wang et al., 2008). Hence, further investigation is required to fully characterize the role of T-cells subpopulations in cancer cachexia.

Metabolic and molecular alterations in skeletal muscle, related to immune dysfunction and systemic inflammation, can occur in patients prior body weight loss (Baracos et al., 2018). Notably, a decline in contact hypersensitivity, a parameter for cell-mediated immunity, has been reported in tumor-bearing pre-cachectic mice, evidencing dysfunction of the immune system preceding weight loss (Faber et al., 2009). Accordingly, the weights of immune-related organs, such as the thymus and spleen, are significantly altered in pre-cachectic mice, with a decrease in the thymus weight and an increase in spleen weight (Eun Ju et al., 2019). The abundance of T-cell populations in spleen is dramatically reduced while cytokines associated to cachexia, such as IL-6, consistently increase in a time-dependent manner starting from 3 days after C26 tumor cells inoculation (Eun Ju et al., 2019).

Collectively, these findings demonstrate that immunological changes are observed prior to weight loss during the pre-cachexia stage, indicating that immunological factors might be promising biomarkers for an early detection of cachexia. Indeed, the immune system seems to be one of the first tissue affected during cancer cachexia and systemic inflammation appears to be a main driver of cancer cachexia. For these reasons, the immune system should receive much more attention in the field of cancer cachexia. By deciphering its role, we might pave the way for innovative therapeutic strategies to counteract both tumor and cachexia progression.

## The adipose tissue: A privileged target in cachexia

The official definition of cancer cachexia states: “a multifactorial syndrome characterised by an ongoing loss of skeletal muscle mass (with or without loss of fat mass) that cannot be fully reversed by conventional nutritional support and leads to progressive functional impairment. The pathophysiology is characterised by a negative protein and energy balance driven by a variable combination of reduced food intake and abnormal metabolism” (Fearon et al., 2011).

Based on this definition, adipose tissue can be unaffected, but most cancer patients and animal models experience loss of fat body mass (Ebadi and Mazurak, 2014). Interestingly, adipose tissue abnormalities precede skeletal muscle wasting (Das et al., 2011; Kir et al., 2014, 2016; Petruzzelli et al., 2022). Indeed, Das et al. (2011) demonstrated that functional lipolysis is essential for the induction of muscle wasting and that knocking-out adipose triglyceride lipase (ATGL) or hormone-sensitive lipase (HSL) is sufficient to totally or partially impair muscle breakdown, respectively. Therefore, adipose tissue and skeletal muscle establish a crosstalk in cachexia development. Lipolysis is triggered by different stimuli, such as pro-inflammatory cytokines and tumor-derived Zinc-α2-glycoprotein (ZAG) (Ebadi and Mazurak, 2015), inducing the release of free-fatty acids in circulation. The excessive oxidation of free-fatty acids in skeletal muscle, induced by several tumor-derived factors, seems to be among the earliest events in prompting muscle wasting (Fukawa et al., 2016), supporting a functional and chronological link between lipolysis and muscle breakdown.

Adipose tissue, in particular the white compartment (WAT), is affected in cachexia also the browning process that is mediated by the upregulation of mitochondria content (Porporato, 2016). During cachexia progression, WAT browning is associated with lipid mobilization and increase of the expression of Uncoupling protein 1 (UCP-1), also called thermogenin, contributing in this way to the dissipation of energy through heat production (Petruzzelli et al., 2014). Therefore, WAT browning massively contributes to resting energy expenditure (REE), worsening negative energy balance and body wasting during cachexia development. Evidences from mouse model of pancreatic cancer suggest that adipose browning precedes adipose tissue wasting (Kordes et al., 2021) and, consistently, expression of UCP1 and body temperature are both increased in pancreatic cancer patients before cachexia manifestation (Kordes et al., 2021). Interestingly, Spiegelman lab demonstrated that the tumor-derived parathyroid-hormone-related protein (PTHrP) induces adipose tissue browning and muscle wasting (Kir et al., 2014) and that the knocking-out of PTH-receptor only in adipose tissue results not only in impaired browning process but also in preserved muscle mass (Kir et al., 2016).

Adipose tissue can contribute to cachexia development also through the release of adipokines. Adiponectin is upregulated in cancer patients, irrespectively of cachexia manifestation, and resistin does not display differences between non-cachectic or cachectic patients. In contrast, leptin appears to be the most interesting adipokine in the context of cachexia. Leptin is a hormone released by adipocytes and enterocytes in small intestine impairing hunger feeling. The circulating level of leptin has been reported to be reduced in cachectic patients, with a direct association with appetite and insulin resistance, which are two other hallmarks of cachexia syndrome (Smiechowska et al., 2010). In cachexia, the appetite regulation by leptin is impaired, and its role in insulin resistance can be important in worsening catabolic status of skeletal muscle.

In conclusion, these observations indicate that adipose tissue abnormalities functionally and temporally precede skeletal muscle wasting, leading to depict a hypothetical timeline in which, starting from pro-inflammatory environment, browning anticipates lipolysis that, in turn, precedes muscle wasting.

## Pancreas: Dual effects in cachexia progression

Pancreatic cancer is the most associated with cachexia development, reaching over 80% of patients (Fearon, Glass and Guttridge, 2012; Baracos et al., 2018). This close association suggest a key role of pancreas in the molecular mechanisms underlying cachexia syndrome. Physiologically, pancreas exerts both endocrine and exocrine functions: on one hand it regulates glucose homeostasis through insulin and glucagon (endocrine pancreas), on the other hand it secretes enzymes necessary to nutrient digestion and uptake in the gut (exocrine pancreas). Up to 90% of patients with tumor at the head of pancreas experience an insufficiency of exocrine compartment during the progression of disease, negatively influencing nutrient uptake and thus resulting in malnutrition and several deficiencies (Kordes et al., 2021). Concerning the endocrine functions, about 75% of patients with pancreatic cancer experience glucose intolerance or diabetes (Muniraj and Chari, 2012). Interestingly, an impaired glucose tolerance is frequent in general in cancer patients, representing one of the first metabolic derangement upon tumor growth. Indeed, the high glucose demand of cancer cells induces a general adaptation of tissues, in particular skeletal muscle and liver, to finally increase glucose production. The elevation of glucose in the circulation leads to insulin synthesis that, when overproduced, finally results in peripheral insulin resistance, affecting adipose and muscle mass (Masi and Patel, 2021). Consistently, insulin resistance is a common feature in cachectic patients and animals (Tisdale, 2009; Honors and Kinzig, 2012). However, insulin resistance often improves after surgery in patients affected by pancreatic cancer, but not the exocrine insufficiency, strongly highlighting pancreatic exocrine function as a crucial clinical issue (Wu et al., 2013; Kang et al., 2016; Maignan et al., 2018). Finally, heterotopic transplantation of pancreatic cancer cells does not induce cachexia, while orthotopic pancreatic cancer triggers cachexia that can be reduced with enzymes replacement, enforcing the crucial role of exocrine pancreas functions in pancreatic cachexia development (Kordes et al., 2021).

Although the insulin resistance results to be necessary and sufficient to induce body wasting in Drosophila (Figueroa-Clarevega and Bilder, 2015; Kwon et al., 2015), its role in cachexia development appears controversial in mammalians. Indeed, insulin deregulation is a common host adaptation to cancer growth, but cachexia significantly interests only a variety of cancer types. It is important to note that insulin resistance observed in cancer is different to what is observed in type-2 diabetes, being characterized by normal fasting glucose level associated with any insulin level (Dev, Bruera and Dalal, 2018). Moreover, the weight loss consequence of metabolic changes due to type-2 diabetes often normalize glucose control, in contrast to what observed in cancer patients (Sah et al., 2013). In C26-bearing mice, cancer cells transplantation is sufficient to trigger insulin resistance before cachexia onset, and its improvement with rosiglitazone results in reduction of cachexia early markers (Asp et al., 2010). Accordingly, insulin administration relieves cachexia symptoms in cancer patients (Lundholm et al., 2007). Similarly, administration of metformin, the main first-line medication for the treatment of type 2 diabetes, reduces muscle wasting in tumor-bearing rats (Oliveira and Gomes-Marcondes, 2016), but metformin induced opposite results in a phase-2 trial (Kordes et al., 2015). The differences related to insulin sensitivity in cancer patients with or without cachexia might rely on degree of peripheral resistance as additional mechanisms in cancer cachexia, such as adipose tissue alterations and hormonal deregulations (glucagon, GLP-1, ghrelin, vitamin D, testosterone, apelin), in turn enforce it (Bartlett, Charland and Torosian, 1995; Burney et al., 2012; Guillory, 2013; Borner et al., 2018; Dev, Bruera and Dalal, 2018; Cecconi et al., 2022). Therefore, the establishment of insulin resistance seems to be an important contributor to cachexia development.

In conclusion, a crucial role of both endocrine and exocrine functions of pancreas in cachexia development has been evidenced in patients affected by pancreatic cancer. However, if endocrine functions seem to be a contributor/enhancer of cachexia in pancreatic cancer and other tumors, the role of digestive enzymes seems to exert a more prominent role in this syndrome. Indeed, their deregulations have a direct impact on nutrients uptake, gut functionality, and microbiome, and, importantly, pancreatic enzymes alterations are directly associated to adipose tissue wasting, making it an early event in pancreatic cancer-associated cachexia (Danai et al., 2018). Collectively, pancreatic cancer unveils important indications on key steps of cachexia development. Interestingly, the pancreas can be severely affected also by oral and gut microbiota dysbiosis and, conversely, pancreatic exocrine functions largely influence gut microbiota (Kordes et al., 2021).

## Gut and stomach: Bad allies in cancer cachexia

Gastrointestinal tumors, including the already depicted pancreatic cancer, are the most associated with cachexia development (Baracos et al., 2018), suggesting a strong correlation between the tissues and organs of the gastrointestinal tract and cachexia pathogenesis. However, data about the gastrointestinal tract regulation during cachexia is largely unrepresented. Gut and stomach exert key functions potentially crucial in cachexia development, from nutrient uptake to metabolic regulation, and different abnormalities can occur in the gastrointestinal tissues during the progression of cachexia, such as digestive impairment, gut microbiota dysbiosis and barrier dysfunction. Moreover, both stomach- and gut-released factors can be deregulated, further worsening this complex scenario. All these perturbations can deeply affect cachexia development, inducing poor nutrients uptake, systemic inflammation, and metabolic alterations (Delzenne et al., 2015; Bindels and Thissen, 2016).

The stomach is an important regulator of feeding through the release of the hunger hormone called ghrelin. Anorexia is a crucial contributor to the reduction in nutrient uptake, in addition to gastrointestinal dysfunction, having deep impact on the establishment of the negative energy balance observed in cachexia. Food intake is regulated by specific pathways in central nervous system (CNS) and different hormones and cytokines affect them. Among others, leptin, released by adipose tissue in response to adiposity, inhibits food intake, whereas ghrelin stimulates appetite. In cancer cachexia, leptin levels are reduced while ghrelin levels are increased, most probably as a compensatory mechanism, but both are unable to exert their function, suggesting the establishment of a resistance to these hormones (Engineer and Garcia, 2012; Argilés et al., 2014). However, ghrelin is a hormone with multiple functions in addition to orexigenic activity that are of therapeutic interest for the management of cachexia, including increase of adiposity, induction of positive energy balance, and impairment of muscle wasting through a direct action on skeletal muscle (Porporato et al., 2013; Khatib et al., 2018). Indeed, pharmacological administration of ghrelin or mimetic (Anamorelin) have been demonstrated to counteract cachexia with promising results (Khatib et al., 2018). Although regulatory agencies approved Anamorelin for cachexia syndrome in Japan (Wakabayashi, Arai and Inui, 2020), the Phase-III ROMANA trial unfortunately failed to reach one of its primary endpoints in Europe. Indeed, Anamorelin administration resulted in improvement of skeletal muscle mass but not function (Temel et al., 2016; Prommer, 2017). Beside this failure, Anamorelin remains one of the more promising drug for cancer cachexia so far.

In addition to the stomach, the gut plays key roles in cachexia progression. As model of intestinal cancer-related cachexia, Apc^Min/+^ mice carry a nonsense point mutation in the Apc gene, leading to multiple intestinal polyps and tumors development. Apc^Min/+^ mice display increased gut barrier permeability, whom onset correlates with IL-6 blood levels and cancer cachexia manifestation (Puppa et al., 2011). Similarly, C26-bearing mice display augmented intestinal permeability due to increased claudin proteins, that is consistent with what observed in gastric cancer patients, in which high level of claudin and decreased amount of occludin proteins correlate with gut barrier dysfunction (Jiang et al., 2014; Bindels et al., 2018). The increase in gut permeability leads to bacterial translocation and endotoxemia in severely affected mice, worsening systemic inflammation, which has been also confirmed in cachectic patients (Zhang et al., 2012; Klein et al., 2013). Indeed, lipopolysaccharide-binding protein (LBP), a marker of bacterial translocation, is increased in C26-bearing mice and correlates with cachexia manifestation, whereas it is a predictor of overall survival, appetite, anorexia, and cachexia manifestation in lung and colon cancer patients (Bindels et al., 2018). Interestingly, these alterations are independent of anorexia in the C26 model, and the blocking of IL-6 not only counteracts cachexia progression but also restores microbiota dysfunction (Bindels et al., 2018).

In addition to alterations in gut permeability, a disruption of the intestinal microbiota homeostasis called dysbiosis also occurs in cancer cachexia, affecting its development (Herremans et al., 2019). A direct link between microbiota and skeletal muscle function has been highlighted by treating mice with broad-spectrum antibiotics, which results in reduced muscle endurance and increased fatigue, with no changes in muscle mass or composition, that were restored after natural bacterial repopulation (Nay et al., 2019). Gut microbiota dysbiosis highly affect blood metabolome (Oliphant and Allen-Vercoe, 2019), and, accordingly, restoration of gut microbiota reduces systemic levels of inflammatory cytokines and relieves muscle wasting in mice (Bindels et al., 2012; Varian et al., 2016; Huang et al., 2017). An important question is to understand whether dysbiosis is related to cancer or more specifically to cachexia. To address this issue, Pekkala et al. (2019) demonstrated that C26 cancer alters gut microbiota, but Activin receptor blocker, used as anti-cachectic agent (Zhou et al., 2010), has only marginal effects on microbiota composition.

In summary, alterations in gastrointestinal district plays a key role in cachexia development, as suggested by high frequency of this syndrome in patients with tumors in the gastrointestinal tract, with high impact in nutrient uptake and systemic inflammation. However, it is not clear in which phase of the cachectic syndrome gut dysfunctions occur, despite evidences suggest that alterations in the gut appear at the onset of cachexia. Beside pancreas and gut, liver as component of the gastrointestinal tract has also been reported to be affected in cancer cachexia as described in the following section.

## Liver: Key modulator of energy expenditure

The liver is a metabolic factory serving as both storage and synthesis site for energy substrates such as glucose, aminoacids, fatty acids, but also hormones, Insulin-like Growth Factor-1, and acute phase proteins. Liver mass is a significant predictor of energy expenditure and cachectic patients frequently show an inflamed fatty liver. A retrospective study on patients with colorectal cancer unraveled an increase in liver mass concomitant to elevation of resting energy expenditure (REE) and loss of skeletal muscle and adipose tissue (Lieffers et al., 2009). The largest changes in body composition are predominantly in liver and muscle and occurred between 4.2 and 1.2 months prior to death in the retrospective cohort. Similarly, the activation of the liver acute phase response has been correlated with increased REE in patients affected by pancreatic cancer (Falconer et al., 1994) and it has been suggested that the liver acute phase response can lead to the shift in the priority of protein metabolism between skeletal muscle and liver that might in turn contribute to muscle wasting in these patients (Fearon et al., 1999). The increased production of acute phase proteins by liver contributes to inflammation in patients with cancer and the C-reactive protein (CRP) appears to be an important parameter for prognosis (Proctor et al., 2011). Beside systemic inflammation, it is also important to point out that the number of liver-infiltrating macrophages has been reported to be higher in cachectic patients affected by pancreatic cancer at stages 3 and 4, and to be negatively correlated with nutritional status (Martignoni et al., 2005). Defects in hepatobiliary secretion, mainly due to a process termed “inflammation-induced cholestasis”, and in bile acid metabolism, which in turn fuel hepatic inflammation, have also been reported in cachectic mice (Thibaut et al., 2020, 2021). Accordingly, targeting bile acid metabolism relieves cancer cachexia manifestations in C26-bearing mice (Feng et al., 2021). Overall, these findings point toward an interaction between tumor, immune cells and liver that might play key roles in the progression of cancer cachexia.

Liver accumulates fat and releases low amounts of very low density lipoprotein (VLDL) in tumor-bearing mice (Jones et al., 2012). It has been demonstrated that deregulation in hepatic lipid metabolism results in worsening of cachexia (Seelaender et al., 1998; Jones et al., 2012; Gonçalves et al., 2019). These reductions in fatty acid mobilization and circulating lipids appears to shift liver metabolism towards a more glycolytic phenotype. Indeed, a marked depletion in hepatic glycogen content has been reported in different preclinical models of cancer cachexia (Narsale et al., 2015; Rosa-Caldwell et al., 2019), suggesting a conserved mechanism for alterations in hepatic metabolism across different cancer types. Cachectic liver also shows mitochondrial alterations in mouse models (Rosa-Caldwell et al., 2020), which precede a fibrotic phenotype during the progression of cancer-cachexia also observed in patients (Pinter et al., 2016; Judge et al., 2019). Similarly, it has been reported that liver mitochondria from tumor-bearing rats request a higher amount of nutrients to sustain the ATP production (Dumas et al., 2011). The authors identified alterations in the content and fatty acid composition of cardiolipin as a possible mechanism to explain the hypermetabolism observed during cancer cachexia. Collectively, these data indicate that mitochondrial dysfunction in the liver appears to contribute to alteration in energy metabolism associated with cancer cachexia.

It is now evident that liver dysfunction contributes to cancer cachexia progression, but the underlying mechanisms remain not fully understood. A couple of studies indicate that alterations in liver might intervene very early after tumor growth. Indeed, a decrease in HMG-CoA reductase (HMGR) expression has been observed at 1 week after tumor inoculation in mice (Rosa-Caldwell et al., 2019). HMGR is an enzyme highly expressed in the liver that catalyses the rate-controlling step in cholesterol production, and it is subjected to extensive hormonal and dietary regulation. Accordingly, a study showed that levels of LDL cholesterol and total cholesterol appear to be relevant indicators of cachexia stages irrespective of the presence of metabolic syndrome or lipid-lowering medication (Zwickl et al., 2020). These observations indicate that markers of metabolic dysregulation might be exploited for early detection of cachexia progression. In this context, lipoproteins are promising candidates as these macromolecules circulating in blood can be easily measured in a clinical laboratory.

Beside modulation in HMGR expression, the authors also found a decreased expression of Peroxisome proliferator-activated receptor-γ coactivator-1α (PGC-1α) at 1 week after tumor inoculation (Rosa-Caldwell et al., 2019). PGC-1α is a transcription cofactor that has been shown to be a potent activator of mitochondrial biogenesis. Hence, mitochondrial dysfunction might be an early event in liver upon tumor progression as previously suggested (Dumas et al., 2011; Rosa-Caldwell et al., 2019). Another study has reported alterations in liver prior to the induction of cachexia in ApcMin/+ mouse. The authors found that the expression of ER-stress markers is modulated in liver both before and after cachexia onset (Narsale et al., 2015). More specifically, progression of cachexia reduces the expression of upstream ER-stress markers such as BiP and IRE-1α in liver, while induces its downstream target CHOP (DNA-damage inducible transcript 3).

Altogether, these data unravel that alterations in liver homeostasis can occur before the onset of the cachectic phenotype defined as body and muscle weight loss. Further investigations are warranted to decipher the early alterations in liver homeostasis upon tumor growth and before the onset of the cachectic phenotype to both identify novel biomarkers to predict cachexia and to find novel insights into the mechanisms triggering the cachectic phenotype.

## Brain: Victim or culprit?

The crucial role of anorexia in the progression of cachexia suggests an important contribution of brain in the establishment of cachexia, despite the investigations in this field are very limited. Several reports indicate that appetite loss is an early event in cachexia progression, occurring before muscle wasting (Yeom et al., 2021; Yeom and Yu, 2022). The cause of anorexia is elusive but inflammatory mediators and tumor-derived factors appear to play a key role in the loss of appetite (Argilés et al., 2019a). In particular, systemic inflammation is also associated with inflammation in the hypothalamus, whom nuclei exert a profound effect on energy homeostasis regulation, resulting in the activation of anorexigenic neurons (proopiomelanocortin, POMC, and cocaine-and-amphetamine regulated transcript, CART) and inhibition of orexigenic neurons (neuropeptide Y, NPY, and the agouti-related protein, AgRP) (Argilés et al., 2019a). Pro-inflammatory cytokines induce illness behaviours and are acquired and amplified near the CNS nuclei linked to energy homeostasis regulation (Olson, et al., 2021b). The IL-1β is the main cytokine produced both peripherally and centrally in response to tumor development, but its genetic deletion does not alleviate fatigue signs in different preclinical models, suggesting that IL-1β might be the initial driver or regulator of some symptoms but not the unique therapeutic target (Olson, et al., 2021b). Indeed, other cytokines are highly expressed in the brain, such as IL-6, TNF-α and LIF. For example, TNFα is a strong mediator of CNS inflammation and is likely implicated in the brain-fat axis activation that triggers adipose tissue wasting in cachexia. However, targeting inflammatory cytokines in cachexia fails to properly counteracts the syndrome, suggesting that systemic inflammation might be crucial in the induction of cachexia but not essential for its maintenance (Olson, et al., 2021b).

In addition to inflammation, other factors contribute to anorexia. Beside the already mentioned ghrelin and leptin, also Glucagon-like peptide 1 (GLP-1), Lipocalin 2 (LCN2), Insulin-like 3 (INSL3), and Growth differentiation factor 15 (GDF15) can induce anorexia in cancer cachexia by directly acting on the brain centres of appetite regulation (Argilés et al., 2019a; Olson, et al., 2021b). The latter three factors have been recently reported to act on CNS circuitry to drive cachexia (Olson, et al., 2021b). The stress factor GDF15 is frequently upregulated in cachexia, in both patients and preclinical models, and is implicated in anorexia, adipose and muscle wasting, making it a promising therapeutic target for cachexia syndrome (Sohail Ahmed et al., 2021). Recently, it has been demonstrated that inhibition of GDF15 counteracts cachexia by reducing activation of sympathetic nervous system on adipose triglyceride lipase (ATGL) and hormone-sensitive lipase (HSL) in WAT, highlighting its major contribution in cachexia development besides appetite regulation (Suriben et al., 2020). In contrast, LCN2 deletion improves fat and muscle mass mainly through appetite regulation, whereas INSL3 elevation induces anorexia that precedes adipose tissue wasting that, in turn, anticipates muscle atrophy (Olson, et al., 2021a; Yeom et al., 2021). Finally, anorexia is influenced by several other aspects, such as adverse events related to chemo/radiotherapy, gastrointestinal dysfunctions, alteration in taste perception, reduced motor activity, psychological distress tending to depression (Argilés et al., 2019a). Despite the depicted crucial role of anorexia and nutrient intake in cachexia development, the total parenteral nutrition is largely ineffective in cancer patients (Evans et al., 1985), unveiling the great complexity of this syndrome.

Both human and animals undergoing cachexia display hallmarks of stress such as elevated glucorticoid circulating levels. Stress response induces the evolutionarily conserved fight-or-flight program in CNS, which results in increased metabolic rate and elevated energy expenditure through the upregulation of sympathetic nervous system tone (Olson, et al., 2021b). The activating stressors range from fasting and malnutrition to acute illness and fear, all of them implicated in cachexia at different levels (Olson, et al., 2021b). The glucocorticoid release is mediated by the activation of the hypothalamic-pituitary-adrenal axis by, among others, IL-1β and GDF15 (Argilés et al., 2019a; Cimino et al., 2021). Elevated glucocorticoid levels induce skeletal muscle wasting, increase cardiac contractility, alter liver metabolism, and enforce IL-6-mediated browning of WAT (Argilés et al., 2019a; Kordes et al., 2021; Martin et al., 2022). Indeed, impairing glucocorticoid signalling results in skeletal muscle mass preservation in different cachexia models (Braun et al., 2011, 2013, 2014). Finally, another contributor to cachexia relies on alterations of hypothalamic-pituitary-gonadal axis, which results in decreased testosterone levels in male, reaching to affect 40%–90% of cancer patients (Olson, et al., 2021b).

A last aspect to emphasize in relation to brain involvement in cachexia development is the psychosocial impact related to tumor diagnosis. Depression and anxiety are more prevalent in cancer patients than in global population, and it has been highlighted a positive correlation between pro-inflammatory cytokines and depression (Seruga et al., 2008; Kordes et al., 2021). Cachectic patients are even more associated with depression and anxiety (Sun et al., 2020), further worsening the general scenario of this devastating syndrome. Indeed, depression is in turn a crucial risk factor for anorexia and loss of weight (Kordes et al., 2021).

Despite the temporal aspect of brain involvement in cachexia development is still unknown, the knowledge obtained so far suggests that central activation of stress and anorectic circuitries is an early event, largely dependent on pro-inflammatory cytokines, which anticipates adipose tissue dysfunctions and, in turn, skeletal muscle wasting.

## Biomarkers for cancer cachexia: An unmet medical need

As already depicted, the lack of a consensus for an early diagnosis and management results in inefficacious therapeutic opportunities for cachectic patients. In this sense, identification of early biomarkers could be a breakthrough in the field. Investigating cachexia biology, several molecules have been suggested as possible biomarker candidates, such as inflammatory mediators, circulating factors, metabolites, and microRNAs. The inflammatory mediators can be released both from tumor and host cells and are frequently elevated in cancer cachexia. Among them, IL-6, TNF-α, IL-1β, CRP, and albumin are the most investigated, but the correlation between them and cancer cachexia is elusive in humans (Cao et al., 2021). The Glasgow Prognostic Score, an inflammation-based cancer-prognostic marker composed of serum elevation of CRP and decrease in albumin concentration, predicts patients with systemic inflammation as part of cancer cachexia and has been shown to have independent prognostic value (Laird et al., 2013). Other factors related to the immune system have been proposed as biomarkers such as the neutrophil-to-lymphocyte ratio, which is a prognostic indicator in cancer, and has been recently associated with weight loss and cachexia in a retrospective study in advanced colon, lung, and prostate cancer patients (Barker et al., 2020). Beside inflammatory factors, several other circulating proteins have been linked to cachexia development and thus proposed as potential biomarkers: ZAG is a lipid mobilizing factor promoting WAT browning and lipolysis; Activin A, Myostatin, and GDF15 are TGFβ-family members involved in skeletal muscle atrophy (Activin A and Myostatin) and anorexia and lipolysis (GDF15); PTHrP induces WAT browning and correlates with lower lean body mass in lung and colorectal cancer patients; Angiotensin II induces muscle wasting and is upregulated in cancer patients in both pre-cachexia and cachexia conditions; LPS-binding protein is a marker of gut barrier dysfunction and shows interesting prognostic value for cachexia development and overall survival in patients; Lipocalin 2 regulates food intake and correlates with neutrophil expansion, muscle wasting and survival in pancreatic cancer patients; INSL3 promotes anorexia and its plasma levels correlate with anorexia severity in pancreatic cancer patients (Sanders, Russell and Tisdale, 2005; Fearon, Glass and Guttridge, 2012; Kir et al., 2014; Ebadi and Mazurak, 2015; Loumaye et al., 2017; Loumaye and Thissen, 2017; Bindels et al., 2018; Olson, et al., 2021a; Cao et al., 2021; Sohail Ahmed et al., 2021; Yeom et al., 2021; Talbert and Guttridge, 2022).

Cachexia, being associated with muscle and adipose tissue wasting, displays elevated metabolites deriving from these catabolic processes, such as amino acids, collagen and titin fragments, carnosine dipeptidase 1, glycerol and free-fatty acids (Loumaye and Thissen, 2017). Other metabolites have been recently identified, such as sphingolipids (Morigny et al., 2020) and lipoproteins (O’Connell et al., 2021). Both sphingolipids, lipoproteins, and amino acids seem to be early markers in cachexia development, enforcing the importance of their measurement. Finally, microRNAs and other non-coding RNAs are emerging as novel markers of cachexia with clinical interest (Donzelli et al., 2020; Santos et al., 2020).

The novel omics studies are deepening our knowledge about cancer cachexia (Loumaye and Thissen, 2017; Paccielli Freire et al., 2020; Cui et al., 2022), increasing the list of potential biomarkers for an early diagnosis. By focalizing on both general and cancer-specific cachexia inducing factors, these approaches will allow the development of novel targeted therapies depending on the cancer types (Paccielli Freire et al., 2020). More specifically, metabolomics studies allow to identify non-invasive markers of cachexia such as the already mentioned microRNAs (Donzelli et al., 2020), sphingolipids (Morigny et al., 2020), lipoprotein and amino acids changes (O’Connell et al., 2021), highlighting the crucial role of new technologies in this context (Loumaye and Thissen, 2017; Paccielli Freire et al., 2020; Cui et al., 2022).

Until now, although many potential candidates have been studied, none of these have been validated as an effective clinical biomarker, rendering cancer cachexia even more complex to manage in a clinical context. Many issues need to be addressed and, due to the multifactorial condition of cancer cachexia, a combination of approaches should be considered. Moreover, it is likely not only that cachexia diagnosis will need a combination of different biomarkers and clinical signs, but also that different tumor types will present variable markers, as highlighted by preclinical models. As example, anorexia is present in C26-bearing animals but not in LLC ones, although both tumors induce cachexia (Yeom et al., 2021). Therefore, the mechanisms are multiple and the identification of one ideal biomarker useful for all cancer patients seems to be extremely implausible. Among the cited biomarkers, great attention is currently focused on GDF15, which, in addition to its diagnostic value, is actively under investigation as therapeutic target (Lerner et al., 2015; Suriben et al., 2020; Sohail Ahmed et al., 2021). Hence, much work has still to be done and, until a clear temporal line will not be clarified, the identification of biomarkers able to change the paradigm for cancer patients and their management will be extremely challenging.

## Concluding remarks

**FIGURE 1:**
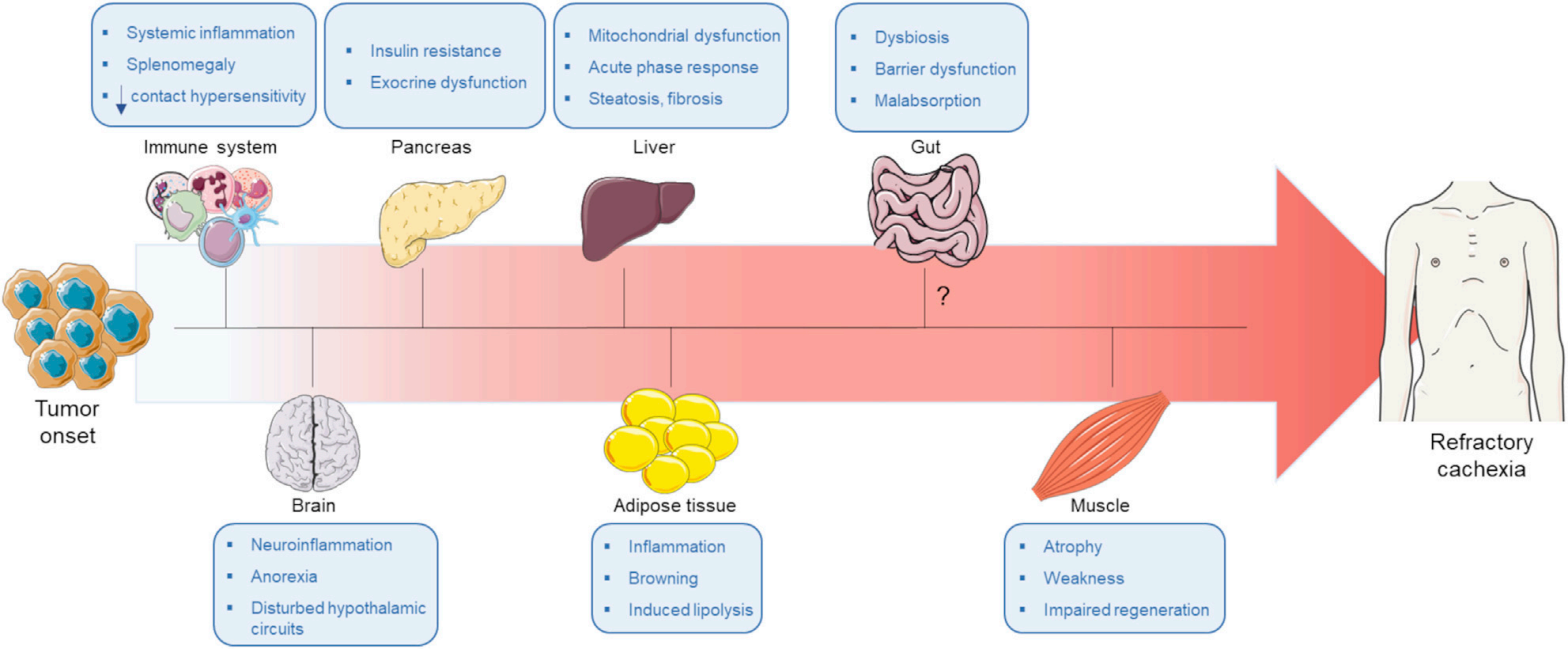
Hypothetical temporal model for cachexia progression by integrating findings from preclinical models and patients. Based on the data reported in literature, we suggest a timeline of organs/tissues derangement upon cachexia progression, highlighting that skeletal muscle wasting, considered as one of the main pathological processes in cachexia, appears to be a late event. In response to tumor growth, the first affected tissue is the immune system, which is a central player in the cachectic process and in the establishment of systemic inflammation. Similarly, several reports indicate that appetite loss is an early event in cachexia progression and although the cause of anorexia is still elusive, inflammatory mediators and tumor-derived factors appear to play key roles in the loss of appetite. Another early event in host adaptation to tumor development is the derangements of both endocrine (insulin resistance) and exocrine functions of pancreas, affecting both tumor growth and cachexia development. Accordingly, pancreatic enzymes alterations have been directly associated to adipose tissue wasting, evidencing that it can be an early event in cancer-associated cachexia. A couple of studies indicate that some alterations in liver, such as mitochondrial dysfunctions and acute phase response, might also intervene early after tumor initiation while steatosis and fibrosis appear later. The most investigated tissues in cachexia, adipose tissue and skeletal muscle, engage a tight crosstalk, with alterations in adipose tissue preceding skeletal muscle wasting. Lipolysis triggered by different stimuli, such as pro-inflammatory cytokines, induces the release of free-fatty acids in circulation. The excessive oxidation of free-fatty acids in skeletal muscle seems to be among the earliest events in prompting muscle wasting, supporting a functional and chronological link between lipolysis and muscle breakdown. Finally, alterations in gut homeostasis have been reported to deeply contribute to cachexia development, but it is not known in which phase of the cachectic syndrome gut dysfunctions occur, despite evidence suggests that alterations in the gut might appear at the onset of cachexia. The Figure was partly generated using Servier Medical Art, provided by Servier, licensed under a Creative Commons Attribution 3.0 unported license.

Despite the large amount of data collected about cachexia over the years, this devastating syndrome remains elusive, and there are still no drug or therapeutic opportunity available for cachectic patients. The parable recently mentioned by Dr. Roeland about the blind men and the elephant, in which each blind person describes the aspect of elephant based on the personal and limited experience by touching the animal (Roeland, 2021), perfectly fits with our knowledge on cancer cachexia. The multifactoriality and complexity of cachexia cannot be addressed by *in vitro* models as they do not reproduce the multiorgan dysfunctions of cachexia and allow to identify only a partial and uncomplete piece of the puzzle. To better understand cancer cachexia, preclinical models are mandatory, but a huge effort should be employed to implement animal models to accurately recapitulate the human phenotype. Indeed, preclinical models have limitations such as the difference of age, with young and health animals employed in the preclinical studies versus the majority of patients in the clinic that are aged and multi-diseased (Talbert et al., 2014; Geppert et al., 2021). Moreover, cachexia should be studied over time, taking in consideration the early signs of cachexia manifestation, to identify the most favourable time window for therapeutic intervention. Indeed, mice or patients prior to weight loss are defined as pre-cachectic, and it is now evident that cachexia starts in other tissues than skeletal muscle or adipose tissues, which appear to be affected at late stage (Figure 1). Therefore, although skeletal muscle wasting, including heart weight loss, is one of the main hallmarks of cancer cachexia, it is not the first tissue affected that might explain the failure of therapeutic approaches targeting muscle wasting as it might represent a point of no return. Cachexia and tumor onsets might be concomitant sometimes, and it is also possible that, in some cases, tumor progression might halt due to failure in cachexia establishment. Hence, more attention should be paid to cancer models without development of cachexia, to rule out all the host adaptions to cancer growth unrelated to cachexia syndrome, and to the different types of cachexia (e.g., cancer cachexia versus septic cachexia) to decipher common and distinct cellular and molecular mechanisms. Concomitantly, measurements of multiple parameters associated with cachexia should be evaluated more deeply in the clinical context, with two main goals: to identify early signs and biomarkers of cachexia to intervene as soon as possible and to collect observations that can be further investigated in preclinical studies to develop animal models closer to human patients. As described in this review, cachexia is a global derangement of multiple tissues, often resulting in a general resistance to multiple factors (such as insulin, leptin, ghrelin, etc.). When multiple circuitries break down, patients come close to a point-of-no-return in which health appears definitely lost and therapeutic interventions appear likely impossible (López-Otín and Kroemer, 2021). The main goal is to direct all efforts to avoid the achievement of this state, focusing on the earliest signs.

In conclusion, many actions are needed to better understand cancer cachexia (Garcia et al., 2022). Clinicians should better take into consideration, beside tumor fighting, also lean and fat body mass, nutritional and psychological status of their patients, and multimodal therapeutic approaches should be considered to counteract this syndrome. A large amount of data stratifying patients is necessary to understand other possible variables in cachexia development, such as age and sex of patients, and to identify early biomarkers. The future in the field of cancer cachexia will be challenging but only a global effort will unveil the underlying mechanism of this devastating syndrome and will allow the identification of therapeutic strategies to improve quality of life and survival of patients.
